# Editorial: Artificial intelligence in human physiology

**DOI:** 10.3389/fphys.2022.1075819

**Published:** 2022-11-18

**Authors:** Chin Siang Ong, Laura Burattini, Stefano Schena

**Affiliations:** ^1^ Division of Surgical Outcomes, Department of Surgery, Yale School of Medicine, New Haven, CT, United States; ^2^ Department of Information Engineering, Università Politecnica delle Marche, Ancona, Italy; ^3^ Division of Cardiothoracic Surgery, Department of Surgery, Medical College of Wisconsin, Milwaukee, WI, United States

**Keywords:** artificial intelligence, physiology, cardiovascular, respiratory, endocrine

Although artificial intelligence (AI) has been established as an academic discipline more than six decades ago, rapid advances have arguably been made in the first two decades of the twenty-first century, due to improvements in computer technology, machine learning algorithms, development of general-purpose computing on graphics processing units, increased access to big data and rise in cloud computing, amongst many other reasons. AI has been used to solve a wide range of challenging problems from all aspects of life, such as business, education, security, medicine (Topol, 2019; Rajpurkar et al., 2022) and, of interest in this collection, human physiology. The purpose of this collection is to broadly highlight recent advances made by applying AI to various biological systems in human physiology, in particular the cardiovascular, respiratory and endocrine systems.

Four articles greatly showcase the application of AI towards the solution of previously known diagnostic limitations of the cardiovascular system. In the field of ischemic myocardial disease, Zhao et al. presents several models based on support vector machine, using calculated sample entropy, spatial heterogeneity index and temporal heterogeneity index of ST-T segments of electrocardiograms (ECGs) and vectorcardiograms, as input features, with the combined model having the best classifier performance as a non-invasive tool for detection of myocardial ischemia. When applying AI in the interpretation of structural heart disease, Bailoor et al. trained a linear discriminant classifier using “acoustic signatures” of healthy and stenotic aortic valves based on principal components of heart sounds and valve status in order to detect aortic valvular anomalies. Along the path of electrocardiographic diagnostics and arrhythmia interpretation, Brisk et al. demonstrated how wave segmentation can be a useful form of ECG representation learning, which improves model performance on downstream tasks. Finally, Cámara-Vázquez et al. discussed the potential of deep convolutional neural networks and body surface potential mapping to identify target regions for ablation in patients with atrial fibrillation.

The collection includes two articles about the respiratory system. Giri et al. wrote a perspective article about how data from pulmonary function tests (PFTs) have potential to be used for both supervised learning and deep learning, notwithstanding the challenges the authors thoughtfully laid out. Meanwhile, Chen et al. proposed an objective measure to quantify the late-expiratory flattening of the flow-volume loop, which is currently visually inspected by clinicians. They found that an angle made by two selected linear fitted lines improved inter-rater agreement on the presence of the late expiratory flattening and may help in the assessment of small airway disease in PFT interpretation.

Finally, in the endocrine system, Zulfiqar et al. showed results of a pilot study, applying a remote monitoring platform to a small cohort of COVID-19 patients, using data from multiple non-intrusive medical sensors combined with an inference engine with evidence-based rules, to monitor dysglycemia *via* generated alerts. Ilari et al. meanwhile, used three supervised machine learning algorithms to determine factors that determine progression of patients with gestational diabetes mellitus to type 2 diabetes mellitus.

These models have great potential in assisting physicians, especially in busy clinical settings, such as emergency departments where timing, in terms of seconds, is of the essence in addressing acute myocardial ischemia, and human interpretation alone may miss important diagnoses. It can also be used in less urgent settings, such as inpatient or outpatient settings to assist cardiologists in making diagnoses of aortic valve disease and guide elective atrial fibrillation ablation strategies, or pulmonologists in diagnosing small airway disease and other respiratory diseases. Such AI models also find application in remote settings, for monitoring *via* telemedicine of patients with difficult blood glucose control or used to predict disease progression in gestational diabetes. It will be desirable if these models can be implemented and investigated as novel devices in randomized clinical trial settings, to assess their actual impact in clinical care.

AI is also scalable and can screen large amounts of structured tabular data such as blood glucose level readings, and unstructured data such as ECGs, recorded auscultated heart sounds, or pulmonary function tests, in a high-throughput fashion, to alert physicians of possible underlying anomalies in the circulatory, respiratory, or endocrine systems. In these cases, each AI-generated interpretation should be generated before the physician signs off on the investigation, so that it is available at hand before committing to diagnostic conclusion. Caution needs to always apply, as trained models can also bias physicians and lead to undesirable heuristic shortcuts. Thus, it is largely advisable that a physician continues to interpret diagnostics at hand without AI support first, before gaining access to the above proposed AI interpretation. We must also be mindful that if AI-generated interpretations are frequently inaccurate or uninterpretable, or if AI-generated alerts are frequently inaccurate or excessive, this can lead to alert fatigue which can desensitize healthcare providers and lead to ignored interpretations or missed alerts, which will subsequently lead to delayed action or non-response. More importantly, it should always be remembered that human bedside physician-patient personal interaction remains crucial and must not be neglected due to the availability of AI model results (Ong and Lawton, 2021).

In conclusion, we hope this collection has demonstrated thematically to readers, over multiple biological systems in human physiology, how AI can integrate with traditional diagnostic tests in medicine, and how AI can sometimes determine additional findings missed by traditional diagnostic tests. AI continues to be an exciting area of development in the field of medicine and has great potential to change the way medicine is practiced and the understanding of human physiology, in the next decade and beyond.
